# A Surfactant Directed Microcrystalline Cellulose/Polyaniline Composite with Enhanced Electrochemical Properties

**DOI:** 10.3390/molecules23102470

**Published:** 2018-09-26

**Authors:** Mahnaz M. Abdi, Paridah Md Tahir, Rawaida Liyana, Ramin Javahershenas

**Affiliations:** 1 Young Researchers and Elite Club, Islamshahr Branch, Islamic Azad University, Islamshahr 3314767653, Iran; 2 Institute of Tropical Forestry and Forest Products, Universiti Putra Malaysia, Serdang 43400, Malaysia; parida@upm.edu.my (P.M.T.); rayda_beep91@yahoo.com (R.L.); 3 Department of Organic Chemistry, Faculty of Chemistry, Urmia University, Urmia 5756151818, Iran; jshbco@yahoo.com

**Keywords:** conducting polymers, microcrystalline cellulose, chemical polymerization, modified electrode

## Abstract

In this study a cationic surfactant, cetyltrimethylammonium bromide (CTAB), was used as a soft template for in situ chemical polymerization of aniline on the surface of microcrystalline cellulose (MCC). The morphology of the wire-like and porous nanostructure of the resulting composite was highly dependent on the MCC and CTAB concentrations. The effect of the MCC and CTAB concentrations on the electrochemical and morphological properties of the polyaniline (PAni) nanocomposite was studied. Cyclic voltammograms of modified PAni/MCC/CTAB electrode displayed a high current response and the effect of scan rate on the current response confirmed a diffusion controlled process on the surface of the electrode that makes it suitable for sensor applications. The overlapping characteristic peaks of pure PAni and MCC caused peak broadening at 3263 cm^−1^ in the IR spectra of PAni/MCC/CTAB nanocomposite that revealed the interaction between NH of PAni and OH group of MCC via electrostatic interactions. The addition of MCC to PAni through chemical polymerization decreased the thermal stability of composite compared to pure PAni. Lower crystallinity was observed in the XRD diffractogram, with 2 theta values of 22.8, 16.5, and 34.6 for PAni/MCC, confirming the formation of PAni on the MCC surface.

## 1. Introduction

Natural cellulose fibers contain crystalline and amorphous domains. Microcrystalline ellulose (MCC) can be synthesized by different processes such as reactive extrusion, enzyme mediated, and acid hydrolysis in that way the amorphous regions are removed and the crystalline domains remain. The hydroxyl groups covering the cellulose surface and orderly arrangement of molecules in MCC allows the cellulose to react well with a variety of materials, including conducting polymers [1]. There are numerous composites of cellulose and its derivatives with synthetic polymers and biopolymers that have been used frequently in different applications such as biomedical applications [2], sensors and actuators, [3] and nanocomposites with good tensile strength [4].

Conducting polymers are some of the most studied materials that have been used to modify the crystals and fibers of cellulose. The poor solubility and dispersibility of conducting polymers in common organic solvents are limiting factors for them to be used in different applications [5,6]. On the other hand the electron transfer in a bulky polymer is relatively slow which limits its application in sensors. A composite of conducting polymers and hydrophilic cellulosic materials with high surface area, tensile strength and good water-dispersibility can overcome these problems [7].

The nanocomposites of cellulose and conducting polymers synergistically combine the electronic characteristics of the conducting polymers with the structural advantages of biobased cellulose, making them useful for different applications [8]. The good interaction between binding sites on the cellulose and cationic species make cellulose a good candidate for sensor devices. Permselective membranes for different species can be modified by a treatment process through changing the surface functionality of cellulose and its permselective properties [9]. A composite of microfibrillated cellulose (MFC) and PPy was prepared by chemical polymerization of pyrrole on the MFC in a hydrogel form [10]. The MFC/PPy nanocomposite with an open, porous structure of intertwined fibers exhibited an acceptable ion-exchange capacity for chloride ions. In recent year, the combinations of PAni and cellulose-based materials have been prepared by various methods to enhance phe hysical and structural properties of the resulting composites. Intrinsic conducting polymers and conductive nanocrystalline cellulose composite have been used as mediators to facilitate electron transfer between the electrode and analyte in biosensors [4,11]. In a study by Lee [4], crystalline nanocellulose-polpyrrole (CNC-PPy) was cast on a microfabricated interdigitated electrode and used as a glucose micro-biosensor. They showed that the compatibility of cellulose with the enzyme combined with the change in the resistance of the doped-PPy could enhance the sensitivity factor by as much as 20.

In many studies composites of PAni/cellulose with enhanced physical and electrochemical properties were used in high capacity biodegradable batteries [1,12,13], chemical sensors [14], and electroactive paper [15]. In biosensor applications, crystalline nano-cellulose (CNC) was introduced into the polymer structure to provide a larger active surface area and higher specific strength [16].

A cholesterol biosensor based on polyaniline and gold nanocomposite was successfully fabricated using a seed-mediated method [12]. Liu et al. [17] engineered a flexible and electrically conductive nanocellulose-based polyaniline composite film. They reported that the composite film with a thickness of 50 µm could be bent up to 180 degrees without breaking.

In the current research we used the cationic surfactant cetyltrimethylammonium bromide (CTAB) as a soft template for aniline/MCC polymerization. There are some reports on the synthesis of conducting polymer nanostructures using surfactants as hard or soft templates to produce a porous material for sensing applications [18]. Using surfactants as template for making nano- and mesostructures can increase the penetration rate of the target molecules into the sensing area and decrease the response time in biosensors [19]. The effect of the CTAB and MCC concentration on the physical and electerochemical properties of the polymer was investigated to develop a nanocomposite with enhanced electrochemical properties for biosensor applications.

## 2. Results and Discussion

### 2.1. Electrochemical Properties

**Figure 1:**
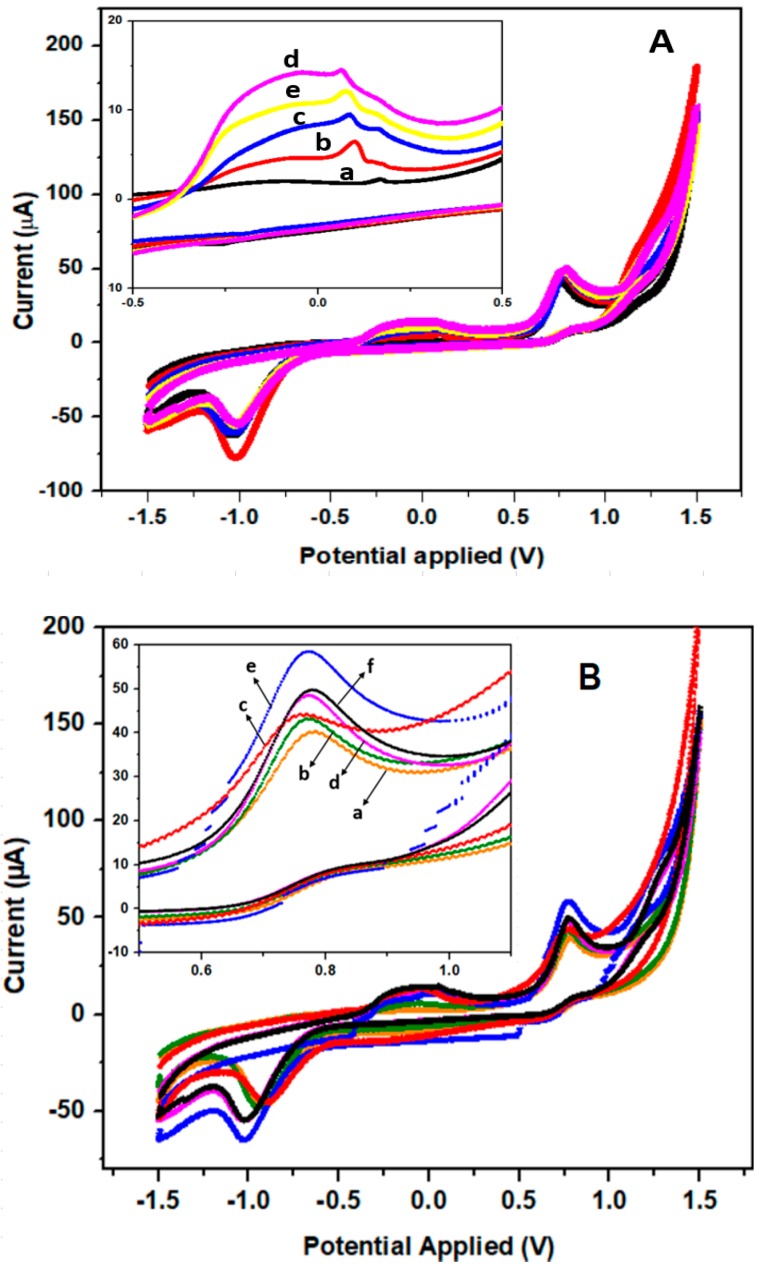
(**A**) Cyclic voltammograms of (a) pure PAni, PAni/MCC nanocomposite with mass ratio of Ani/MCC (b) 0.37/0.63, (c) 0.56/0.44, (d) 0.74/0.26, (e) 0.93/0.07, (**B**) Cyclic voltammograms of PAni/MCC/CTAB nanocomposite prepared from solutions containing different concentrations of CTAB ranging from: (a) 0 cmc (PAni/MCC), (b) 4 cmc, (c) 6 cmc, (d) 8 cmc, (e) 10 cmc (f) 12 cmc. Inset: enlarged anodic peak.

Cyclic voltammetry was done to optimize conditions for nanocomposite preparation and find a composition with the highest anodic current. The effect of MCC and CTAB concentration on the electrochemical properties of nanocomposite was determined using CV performed from −1.5 to +1.5 V at a scan rate of 100 mV s^−1^. Figure 1A depicts the cyclic voltammograms for pure polyaniline and the PAni-MCC composite prepared from solutions containing different Ani/MCC mass ratios. The cyclic voltammogram of the PAni-modied electrode showed anodic peaks at the potentials of 0.13 and 0.81 V. A small oxidation peak observed at 0.13 V is associated with the conversion of the fully reduced leucoemeraldine base to the partially-oxidized emeraldine. As the potential increased the conversion of the emeraldine form to the fully oxidized pernigraniline form occurred at 0.81 V, which is in agreement with the results reported by others [20]. The reduction of the electrically conductive emeraldine (EM) to the non-conducting leucoemeraldine (LEM) was observed at −1.01 V.

The PAni-MCC nanocomposite-modified electrodes presented voltammograms with higher cathodic and anodic peaks (Figure 1A).

It is known that PAni is conductive only in acidic media; however, PAni composites must be able to be used in neutral media as biosensors. On the other hand it has been shown that the ionization of the carboxyl groups of cellulose in a neutral medium will produce an excess net negative charge on the PAni/cellulose composite. In order to compensate the extra negative charges the composite undergoes protonation which increases the proton concentration inside the composite compared to the external solution; consequently PAni/cellulose composite shows conductivity even under neutral conditions [21]. As it can be seen from Figure 1A, by increasing the MCC content (mass ratio of Ani/MCC from 0.93/0.07 to 0.74/0.26) in the nanocomposite, the oxidation peaks at the potentials of 0.13 and 0.81 V increased and the composition of 0.74/0.26 mass ratio (0.16 M aniline) exhibited the highest current response of 14.4 and 49.7 µA, respectively. This was possibly due to the protonation of PAni in the presence of a higher amount of MCC. Another possible reason is due to the capability of MCC to produce nanostructures with a higher surface area and porosity that facilitate the electron transfer. The fibrous and porous structure of PAni/MCC (compared with bulky PAni) which was observed in FESEM images justified a higher electron transfer of the composite. After the addition of MCC content accounting for more than 26% of the composite, the cyclic voltammogram of PAni/MCC presented a lower anodic peak and the nanocomposite with a mass ratio of 0.37/0.63 (0.08 M aniline) exhibited the lowest redox current. This was probably due to the insulating nature of cellulose that restricted the electron transfer in the polymer chain. All voltammograms were taken in 0.1 M PBS of pH 7.0 with a potential scan rate of 100 mV s^−1^ at room temperature.

CTAB content optimization was further conducted to study the effect of CTAB on the electrochemical properties of the nanocomposites. Figure 1B shows the voltammograms of PAni/MCC/CTAB nanocomposite prepared from solutions containing different concentrations of CTAB ranging from 4–12 cmc. The concentration of Ani was 0.16 M and the mass ratio of Ani/MCC was kept fixed at 0.74/0.26. 

**Figure 2:**
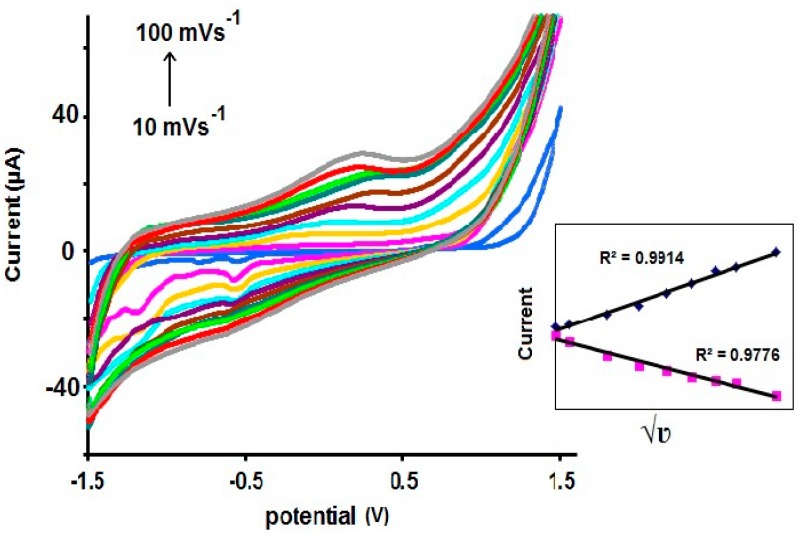
Cyclic voltammograms of PAni/MCC/CTAB-modified electrode with different scan rate. Inset: plot of oxidation and reduction peak current against square root of scan rate. All voltammograms were taken in 0.1 M PBS of pH 7.0 with a potential scan rate of 100 mV s^−1^ at room temperature.

The enlarged anodic peak is displayed in the inset graph for more clarification. The nanocomposites presented an increment in the anodic peaks after addition of CTAB and the nanocomposite prepared from 10 cmc of CTAB showed the highest redox current of 58.7 µA. It was found that CTAB has a significant effect on the formation of the nanostructure of the PAni composite which is related to its function as a soft template for the polymerization of aniline by the self-assembly of the cationic surfactant and monomer. It has been shown that the type of surfactant and its concentration play important role in controlling the diameter of nanoparticles by partially solubilizing the template [22]. Figure 2 shows CVs of PAni/MCC/CTAB-modified electrode with different scan rate. The nanocomposite was prepared from a solution containing Ani/MCC with mass ratio of 0.74/0.26 and 10 cmc CTAB. As it can be seen that both the oxidation and reduction peaks increased with the increasing scan rate. Most cycles showed both the oxidation and reduction peaks, in which the anodic potential shifted towards the lower potential and the cathodic potential slightly shifted to the more negative value as the scan rate increased. The concentration profile around the electrode is influenced by the rate of potential scanning. This phenomenon can change the rate of charge transport indicating the diffusion controlled nature of a system. The anodic and cathodic peaks were found to be proportional to the square root of the scan rate, which clearly indicated the occurrence of a diffusion-controlled process (inset plot) [23].

### 2.2. Chemical, Morphological and Structural Characterizations

#### 2.2.1. Fourier Transform Infrared (FTIR) Spectroscopy

**Figure 3:**
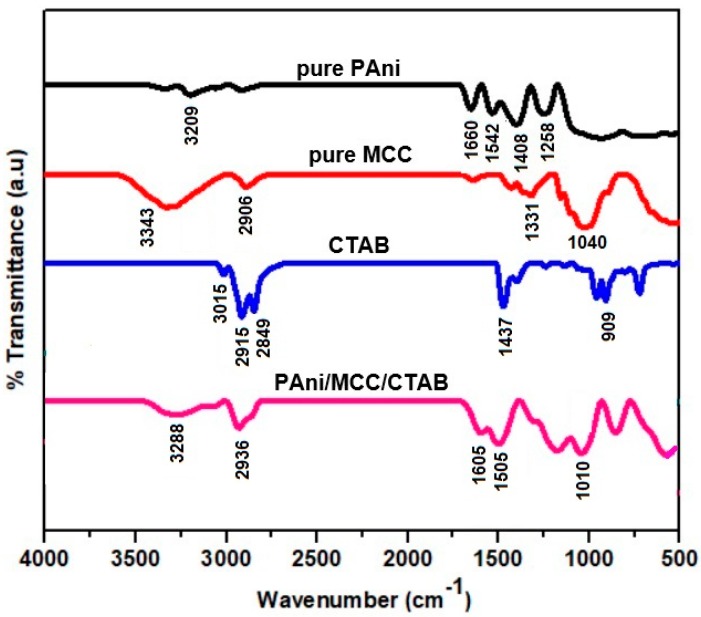
FT-IR spectra of pure PAni, pure MCC, CTAB, and PAni/MCC/CTAB nanocomposite.

Figure 3 compares the FT-IR spectra of pure PAni, MCC, and PAni/MCC/CTAB nanocomposite. Pure doped-PAni showed characteristic peaks at 3209 cm^−1^, 2938 cm^−1^ associated with the N-H stretches and C-H stretch, respectively. The characteristic peaks assigned to the C=C stretches and C=N bond in the polyaniline units appeared at 1660 cm^−1^ and 1542 cm^−1^, respectively. The aromatic stretching and C-C bending vibrations appeared at 1408 cm^−1^ and 1258 cm^−1^ [24,25]. In the MCC structure, a strong O-H stretching absorption band occurred at 3343 cm^−1^ and the peak appearing at 2906 cm^−1^ represented the aliphatic C-H stretching. The sharp peak of C-O stretching of the primary alcohol group (-CH_2_OH) appeared at 1040 cm^−1^, whereas the peak at 1331 cm^−1^ showed the C-O bending. The spectra of PAni/MCC/CTAB nanocomposite (prepared from Ani/MCC mass ratio = 0.74/0.26 and 10 cmc surfactant) is also presented in Figure 3. The spectrum revealed both characteristic peaks of pure PAni and MCC, thus demonstrating the polymerization of aniline on the surface of MCC. The overlapping characteristic peaks of pure PAni at 3200 cm^−1^ and MCC at 3331 cm^−1^ caused peak broadening at 3288 cm^−1^ in the IR spectra of PAni/MCC/CTAB nanocomposite revealing the interaction between NH of PAni and OH group of MCC via electrostatic interactions [26,27]. The nanocomposite of PAni/MCC/CTAB showed lower O-H band intensity compared to the pure MCC, which might due to the crystalline cellulose being covered by PAni, which is in agreement with other studies [28,29]. In the CTAB molecule the CH_3_ asymmetric stretching (ν_as_) and CH_3_ symmetric stretching (ν_sym_) frequencies were observed at 2915 and 2849 cm^−1^, respectively. The N–CH_3_ stretching appeared as a weak shoulder at 2949 cm^−1^. The presence of a peak at 2936 cm^−1^ in the nanocomposite confirmed the existence of CTAB in the composite structure. The C–N stretching vibration observed at 909 cm^−1^ in CTAB shifted to 1010 cm^−1^ in PAni/TA/CTAB nanocomposite [19,30].

**Scheme 1:**
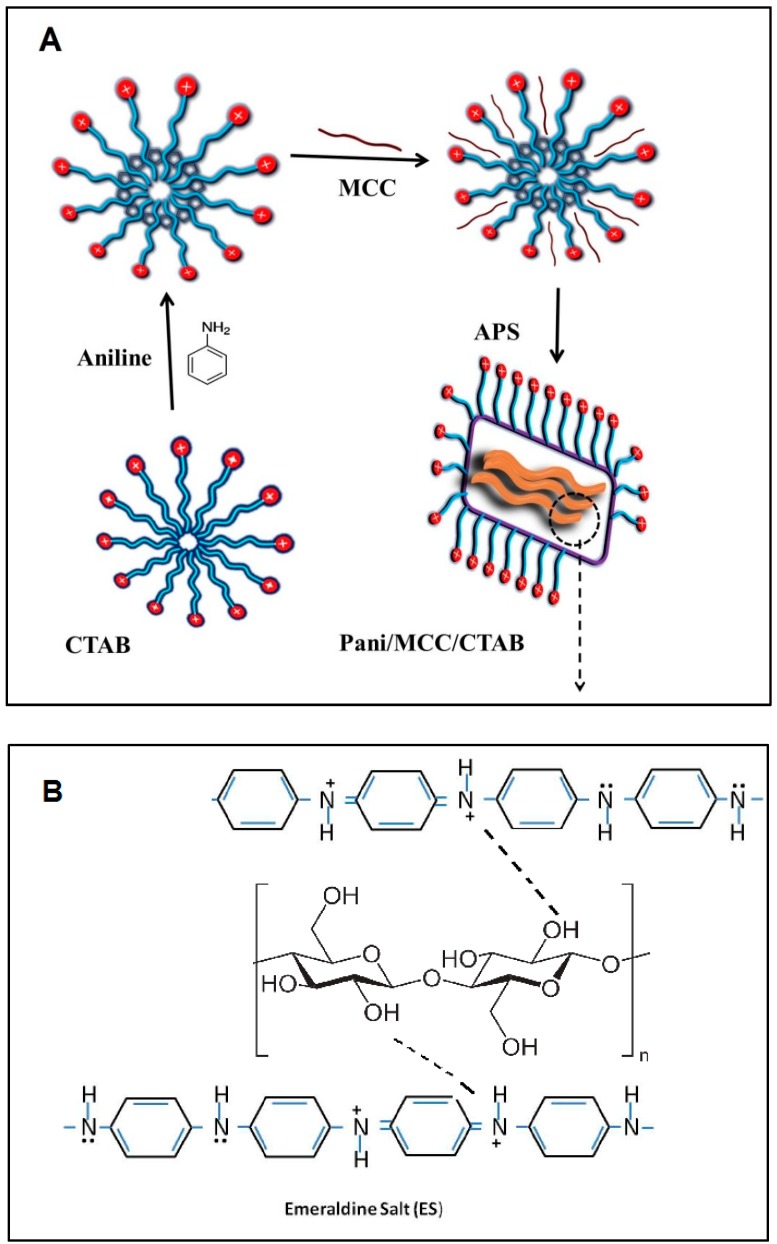
(**A**) View of chemical polymerization of aniline in the presence of MCC and CTAB and (**B**) possible intraction between cellulose and PAni.

The stretching vibration of the benzoid form of PAni appearing at 1505 cm^−1^ in the PAni/MCC/CTAB nanocomposite showed a uniform formation of PAni on the nanofibrous structure. Based on the combined FTIR results, the following structure (Scheme 1) is proposed for PAni/MCC/CTAB nanocomposite.

#### 2.2.2. Morphology

**Figure 4:**
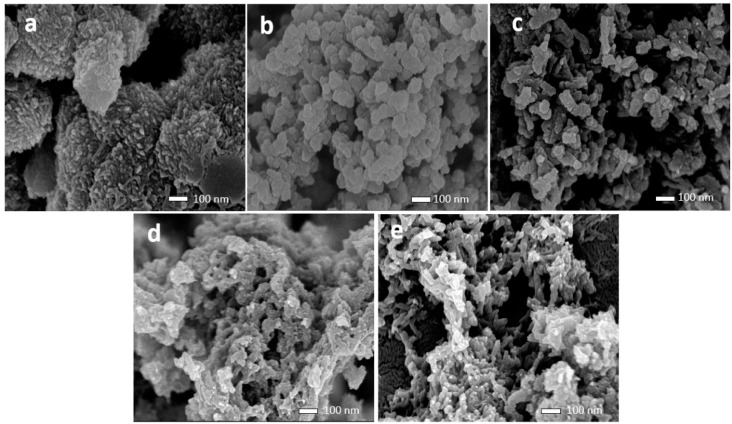
FESEM micrographs of (**a**) pure PAni, and PAni/MCC nanocomposite prepared from solutions containing different mass ratio of Ani/MCC of (**b**) 0.37/0.63; (**c**) 0.56/0.44; (**d**) 0.74/0.26; (**e**) 0.93/0.07.

FESEM micrographs of pure PAni and PAni/MCC with different MCC content are presented in Figure 4. Pure PAni showed a bulky, granular, and dense structure (Figure 4a) but a remarkable changes from bulky and shapeless structure to spherical particles was observed upon addition of MCC to the polymer (Figure 4b), proving the polymerization of aniline on the cellulose surface [31]. The features of the PAni/MCC composite changed slightly to a rod-like structure on further addition of MCC whereupon the fibers become longer with less agglomeration of particles (Figure 4c). The composite prepared from 0.16 M aniline (Figure 4d) showed a fibrous feature with a high porosity nanostructure, which could be beneficial for biomolecule immobilization. The proper mixing of MCC and PAni combines the nanostructure advantage of biopolymer and the electrical properties of PAni explaining the higher anodic current obtained for the composite. The addition of extra amounts of cellulose (Figure 4e) formed intertwined fibers; with nodular agglomerates of particles in some parts of the nanocomposite. It seems that the composite with higher amount of MCC presented a smooth and uniform surface fiber displaying crystalline and insulating cellulose with lower electroactive property that justifies the lower redox current in the CV graph.

**Figure 5:**
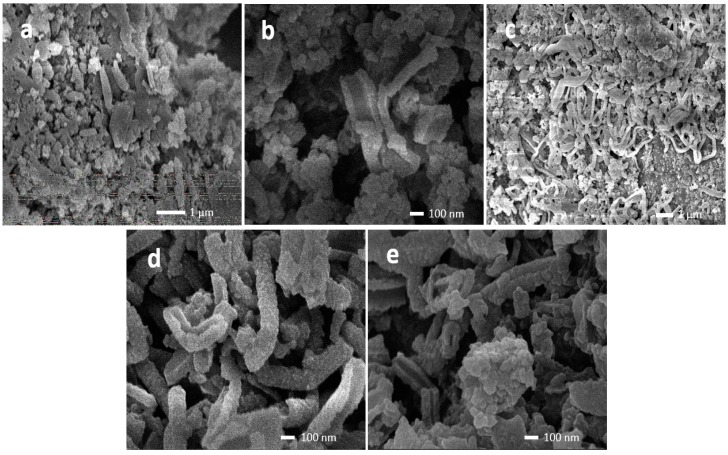
FESEM micrographs of PAni/MCC/CTAB nanocomposite prepared from different concentration of CTAB (**a**) 4 cmc; (**b**) 6 cmc; (**c**) 8 cmc; (**d**) 10 cmc; (**e**) 12 cmc.

Figure 5 presents the morphology of PAni/MCC/CTAB prepared from solutions containing 0.16 M aniline (Ani/MCC mass ratio of 0.74/0.26) and different concentrations of CTAB. After adding 4 and 6 cmc surfactant, a few particles with wire-like structure were observed (Figure 5a,b). It has been proved the cationic surfactant of CTAB works as a soft template to tailor the shape of the polymer into desired nanostructures in such a way that above a critical concentration of surfactants, they assemble into micelles, spherical or cylindrical structures [32]. In our study, by increasing CTAB to t8 and 10 cmc, intertwined wire and ribbon-like structures were observed (Figure 5c,d) providing more porous structures with less agglomerated particles which could facilitate the electron transfer between electrode and mediator.

At high concentrations of CTAB the ribbon-like particles formed were significantly shorter in length and twisted to produce half tube structures (Figure 5e,f).

#### 2.2.3. X-ray Diffraction Analysis

**Figure 6:**
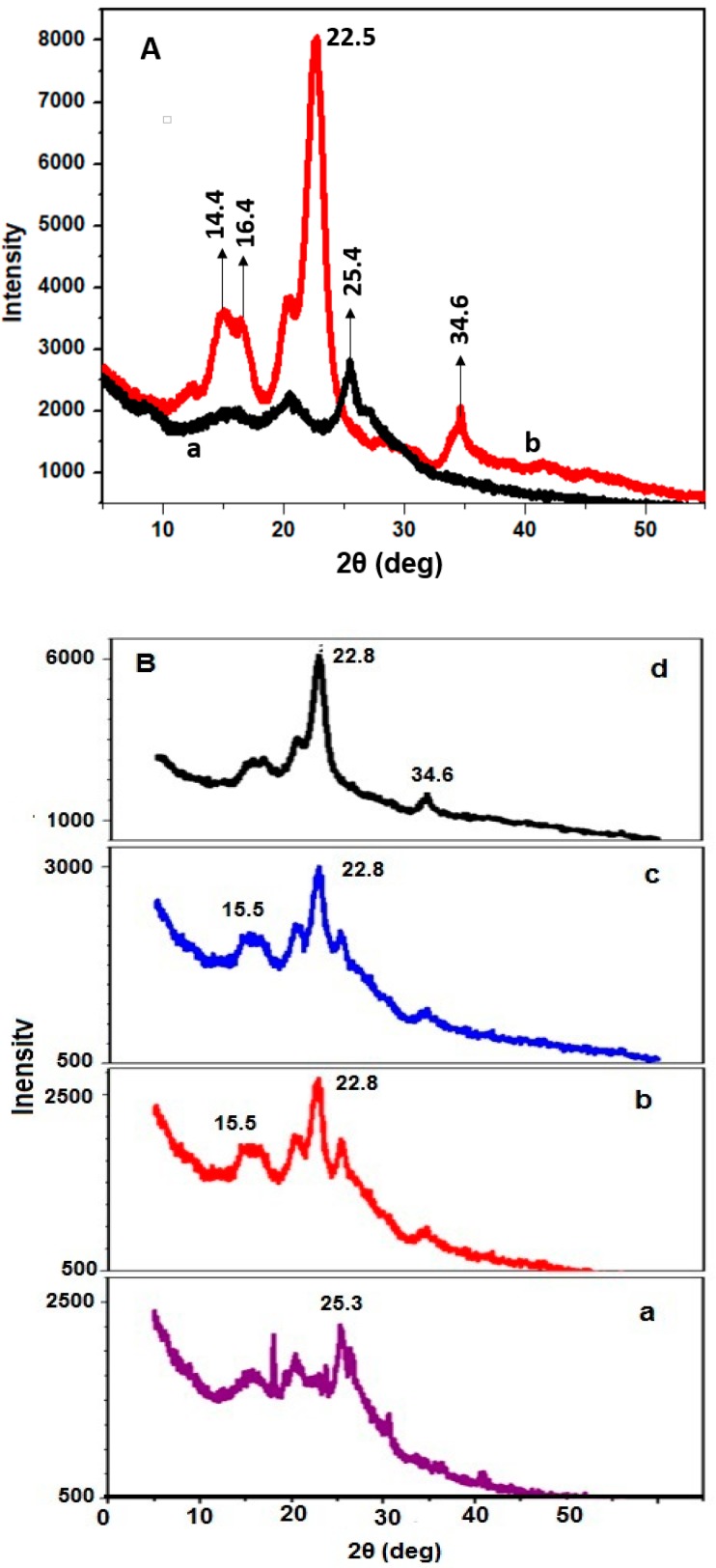
(**A**) X-ray diffractrograms of (a) pure PAni, and (b) MCC; (**B**) PAni/MCC prepared from different mass ratio of Ani/MCC (a) 0.93/0.07; (b) 0.74/0.26; (c) 0.56/0.44; (d) 0.37/0.63.

Figure 6A shows the XRD pattern of PAni, and MCC. Pure PAni presented peaks at 2θ = 20.5° and 25.4°, ascribed to the repetition of the benzoid and quinoid rings in the PAni chains [33] confirming polyaniline was in the form of emeraldine salt [34].

The XRD diffractogram of MCC (Figure 6A(b)) showed the highest intensity at 2θ = 22.5°, and two small peaks at 2θ = 16.4° and 14.6° correspond to the Miller indices of (200), (110) and (1–10), respectively [35]. Pure MCC also displayed a small peak at 2θ = 34.6°, all confirming the typical pattern for cellulose I. 

The effect of MCC content on the crystallinity aspects was studied and Figure 4B compares the XRD diffractograms of PAni/MCC nanocomposites with different mass ratios of Ani/MCC. The nanocomposite with the highest amount of MCC (Figure 6B(d)) exhibited the highest intensity peak at 22.8^o^ and a small peak at 2θ = 34.6°. The nanocomposite with high PAni content (Figure 6B(a)) exhibited the highest peak at 2 theta of 25.3° indicating the superior effect of PAni on the crystallinity of the nanocomposite. The results are in agreement with those obtained by others [17].

#### 2.2.4. Thermogravimetry Analysis (TGA)

**Figure 7:**
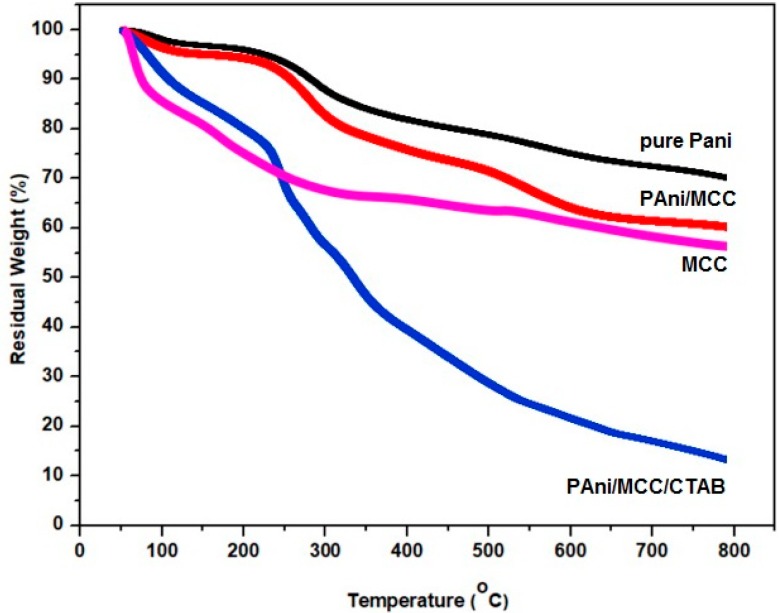
TGA curves of Pure PAni, PAni/MCC, MCC, and PAni/MCC/CTAB nanocomposite.

The TGA plots of pure PAni, PAni/MCC (Ani/MCC mass ratio of 0.74/0.26), and PAni/MCC/CTAB (Ani/MCC mass ratio of 0.74/0.260 and 10 cmc of CTAB) are presented in Figure 7. Doped polyaniline typically exhibits three major weight loss steps. The first weight loss attributed to the removal of moisture starts at 56 °C and continues rapidly up to 150 °C. The second step, due to the dopant decomposition, gradually occurred from 158 °C to 353 °C. The last weight loss happened around 457 °C to 600 °C and is attributed to decomposition of the polymer backbone in agreement with the results of others [36]. The incorporation of crystalline cellulose into the PAni increased the crystallinity of the composite, where more energy was required to evaporate water/acid from the polymer chain [37] resulting a higher degradation temperature for PAni/MCC in the first step. In other words, PAni chains doped with acid in a more crystalline structure would need more energy for removing acid from well arranged polymer chains. Therefore, the second weight loss of PAni/MCC with higher crystallinity shifted to a higher temperature. The pyrolysis of cellulose content in PAni/MCC composite was observed over the range from 230 °C to 350 °C [38] that is higher compared to that of pure cellulose, suggesting the protection of PAni over the surface of cellulose [39].

Pyrolysis of cellulose in microcrystalline cellulose occurred at a higher temperature compared to that of nanocrystalline cellulose [17]. This could be due to the bigger size, higher crystallinity, and higher polymerization degree of MCC.

A lower maximum decomposition temperature was observed for the PAni/MCC nanocomposite compared to that for pure PAni, confirming the interactions between the two constituents of the composite [38]. The thermal stability of PAni was noticeably decreased by incorporating MCC. However, adding CTAB to the composite resulted in lower thermal stability of the nancomposite and PAni/MCC/CTAB nanocomposite decomposed at a lower temperature due to the calcination of CTAB up to 250 °C [40]. CTAB transformations during calcination of CTAB up to ~250 °C is well known and it completely decomposed where there is no trace of CTAB at temperatures from 200 °C–500 °C [40].

## 3. Materials and Methods 

### 3.1. Materials

Microcrystalline cellulose (MCC) was provided by R&M Chemicals (Essex, United Kingdom) and was partially soluble in dilute mineral acids. Aniline used as monomer, purchased from Sigma Aldrich Chemie GmbH (Dramstadt, Germany), was distilled under reduced pressure at 131 °C and stored at 3 °C in the refrigerator until used. Ammonium persulphate (APS) as oxidant, hydrochloric acid (HCl) and phosphate buffer solution (PBS) were provided by R&M Chemicals. Cetyltrimethylammonium bromide (CTAB) was purchased from Fluka BioChemika (Munich, Germany) and a screen printed carbon electrode (SPcE) was obtained from DropSens (Asturias, Spain). All chemicals were of analytical reagent grade and used without further purification.

### 3.2. Preparation of the Nanocomposite and Modified Electrode

Various amounts of aniline were dissolved in hydrochloric acid (HCl) 1M in an ice bath. In a separate conical flask MCC was dispersed in deionized water (DIW) and stirred moderately until the mixture reached homogeneity. Different concentration of CTAB ranging from 4–12 cmc (1 cmc of CTAB = 0.87 mM) was prepared in the DIW and then added into the Ani solution. Then, the Ani/CTAB solution was added into the MCC solution and the mixture was stirred at 0–5 °C for 5 min in the ice bath and left standing for 10 min. The concentration of aniline was adjusted in the range of 0.08 to 0.2 M and the mass ratio of Ani/MCC was selected as 0.37/0.63, 0.56/0.44, 0.74/0.26, and 0.93/0.07 in final mixture. The concentration of CTAB was in the range of 4–12 cmc. Critical micelle concentration, known as cmc, is the concentration of surfactants above which micelles form and all additional surfactants added to the system go to micelles. The solution of APS was added drop wise to the mixture in which the molar ratio of APS and aniline was always kept at 1:1. The mixture was kept stirring for 30 min and let it rest at room temperature for 18 h. The green emeraldine polyaniline nanocomposite (PAni/MCC/CTAB) precipitate was filtered off and washed with distilled water at least 3 times through centrifugation till supernatant become clear. The precipitate was dried in the oven at 60 °C for 6 h. Pure PAni was prepared at the same condition for comparison purpose. Composite of PAni/MCC was prepared in the same manner just without surfactant. 

In order to prepare a modified electrode, 1 mg of the dried sample was dispersed in 2 mL DIW and ultra-sonicated for 15 min to get a stable suspension. To obtain a homogeneous and uniform sample surface on the electrode, 10 μL of suspension was dropped onto the screen-printed electrode (SPE), by drop casting followed by drying at room temperature. 

### 3.3. Methods

The electrochemical measurements were conducted using an Autolab 204 potentiostat connected to a PC and controlled by Nova software version 2.11 (Metrohm, Utrecht, The Netherlands). Field Emission Scanning Electron Microscopy (FESEM) characterization was done by using JEOL JSM-7600F FESEM Microscope purchased from JEOL Ltd. (Tokyo, Japan) using the secondary detector, 7.0 probe size and 5.0 kv acceleration voltages. Powder XRD data were carried out on a PANanalytical EMPYREAN system (Royston, UK) at 4.0 kW power supply, 100 mA current flow and 60 kV operation voltage, and with Cu Kα radiation (λ = 1.54 Å). The thermal gravimetric analysis of samples was done using a TGA/SDTA 851 system (Mettler Toledo, Ohio, USA) under constant heating rate (10 °C/min) and N_2_ atmosphere with the temperature range of 50–800 °C. FTIR analysis was done on the samples by using a Spectrum 100FT-IR spectrometer (Perkin Elmer, Waltham, MA, USA) equipped with Universal Attenuated Total Reflectance (UATR). The nanocomposite (100 mg) was mixed with potassium bromide (KBr) and compressed to form a crystalline, clear pellet and examined in the transmittance mode within a 4000–400 cm^−1^ range.

## 4. Conclusions

In this work it has been shown that the physical and electrochemical properties of polyaniline were enhanced in the presence of MCC and the cationic surfactant CTAB. The SPE electrodes modified by PAni/MCC/CTAB using certain amounts of MCC and CTAB presented the uppermost redox currents that could be appropriate for sensor applications. The PAni/MCC/CTAB nanocomposite prepared from 10 cmc of CTAB showed the highest redox current of 58.7 µA, while PAni/MCC prepared from same composition exhibited an anodic current of 49.7 µA. The lamellar structure between the cationic surfactant and anion of the oxidizing agent, APS, serve them as a template to synthesize nanomaterials with high surface area and porosity. The electrochemical properties of doped PAni combined with the compatibility of cellulose make PAni/MCC composite a good candidate to be used in biodegradable batteries and biosensors. PAni/MCC/CTAB nanocomposite could represent a stable nanostructure platform for enzyme and biomolecule immobilization. The XRD and FESEM results confirmed the formation of s homogeneous PAni composite in the presence of CTAB and MCC
